# The charm of structural neuroimaging in insanity evaluations: guidelines to avoid misinterpretation of the findings

**DOI:** 10.1038/s41398-018-0274-8

**Published:** 2018-10-26

**Authors:** C. Scarpazza, S. Ferracuti, A. Miolla, G. Sartori

**Affiliations:** 1 Department of General Psychology, University of Padua, Via Venezia 8, 35131 Padova, Italy; 2 Department of Psychosis Studies, Institute of Psychiatry, Psychosis and Neuroscience, King’s College London, De Crespigny Park, London, SE5 8AF UK; 3 Department of Human Neuroscience, Sapienza University of Rome, P.le A Moro 5, 00185 Roma, Italy

## Abstract

Despite the popularity of structural neuroimaging techniques in twenty-first-century research, its results have had limited translational impact in real-world settings, where inferences need to be made at the individual level. Structural neuroimaging methods are now introduced frequently to aid in assessing defendants for insanity in criminal forensic evaluations, with the aim of providing “convergence” of evidence on the *mens rea* of the defendant. This approach may provide pivotal support for judges’ decisions. Although neuroimaging aims to reduce uncertainty and controversies in legal settings and to increase the objectivity of criminal rulings, the application of structural neuroimaging in forensic settings is hampered by cognitive biases in the evaluation of evidence that lead to misinterpretation of the imaging results. It is thus increasingly important to have clear guidelines on the correct ways to apply and interpret neuroimaging evidence. In the current paper, we review the literature concerning structural neuroimaging in court settings with the aim of identifying rules for its correct application and interpretation. These rules, which aim to decrease the risk of biases, focus on the importance of (i) descriptive diagnoses, (ii) anatomo-clinical correlation, (iii) brain plasticity and (iv) avoiding logical fallacies, such as reverse inference. In addition, through the analysis of real forensic cases, we describe errors frequently observed due to incorrect interpretations of imaging. Clear guidelines for both the correct circumstances for introducing neuroimaging and its eventual interpretation are defined

## Introduction

Despite the popularity of structural neuroimaging techniques in twenty-first-century research, its results have had limited translational impact in real-world settings, where inferences need to be made at the individual level. Recently, however, a growing number of research groups have attempted to overcome this issue by studying the feasibility of single-case analysis^1–6^.

Structural neuroimaging (sNI) evidence is increasingly being used in criminal trials^7–9^. The literature on sNI and law tends to consider neuroscientific knowledge extremely relevant for assisting in psychiatric assessments of criminal responsibility^10–12^. In the penal field, holding a defendant criminally responsible requires proof that he/she committed the act (*actus reus*) and that the act was intentional, i.e., the defendant committed the act of his/her own free will (*mens rea* or guilty mind)^13^. Crucially, the *mens rea* can be abolished (or weakened) due to the impact of a medical condition on brain functioning^10^, usually a neurologic or psychiatric condition (or, more rarely, organic conditions, such as hypoglycemia^14^). In these cases, the defendant could be considered mentally insane at the moment of the crime and, consequently, not responsible or having diminished responsibility for his/her actions. Thus, the determination of criminal responsibility rests on the evaluation of the mental state of the defendant at the time of the crime^15^.

Critically, a wide body of recent literature has revealed that forensic insanity assessment is particularly prone to different biases^16,17^. Most relevant is the so-called “cognitive bias”, arising from the subjective humanness that influences every step of the forensic psychological assessment^18,19^, from data collection to judicial decisions. These biases arise as forensic experts are human beings with personal knowledge, beliefs, expectations, cognitive schemas, etc. All of these factors influence the way forensic elements are addressed and unconsciously impact the collection, analysis, interpretation and reporting of data^18,20,21^. The hierarchy of expert performance^22^ (HEP) has recently been proposed to better understand expert performance in forensic science and the biases that might arise at each step of forensic work. The HEP has also been applied to forensic psychology and psychiatry^23^, and some bias-mitigating strategies have been proposed^20^. The HEP makes a clear distinction between observations and conclusions, where observations pertain to data observation, collection and analysis, while conclusions depend on the assessment and interpretation of observations^22,23^.

Forensic psychologists and psychiatrists are increasingly aware of biases that may influence expert performances^17,21^. Three critical issues hampering the credibility of forensic experts are as follows: the tendency of experts to be biased by previous expectations^24^, the low inter-rater agreement in conclusions on insanity based on clinical assessments^25–28^ and the variability in the rates of evaluation outcomes as a function of the evaluator^17,28,29^. For these reasons, brain images are now increasingly introduced to assist in the assessment of insanity in criminal cases, with the aim of providing a “convergence” of evidence (including anamnestic, clinical and neuroscientific information). This is of critical relevance, as the *mens rea* of the defendant is pivotal to the judge’s decision^30,31^. The idea is that sNI would provide additional biological data that, when considered in conjunction with classical neurologic, psychiatric and neuropsychologic assessments, would inform the court about the defendant’s responsibility^11^. The literature suggests that sNI evidence should not be used alone and cannot be used solely to explain the cause of a violent crime (e.g., “she killed her mother because she has a frontal lobe lesion”). Rather, sNI findings (i.e., the frontal lobe lesion) should be considered a “hard” correlate of mental disease, symptoms of which (e.g., disinhibition) are causally linked to the crime (e.g., the homicide)^11^. Thus, the criteria for responsibility are currently behavioural and should remain that way^11,12^.

Although the use of sNI in court seems to be a promising advance in reducing the uncertainty of forensic experts’ evaluations^32^, their use is not free from potential risks and biases^11,12,33,34^. One such bias is that neuroimaging results are considered to be objective, which is not always true. In some cases, MRI data need to be analysed with sophisticated techniques^35,36^, and the results can be influenced by different technical choices. This bias concerns the HEP observation level and will not be explored in the current paper. Another worrying concern, at the HEP conclusion level, is the potential problem of the “inferential distance”^37,38^, i.e., the vivid and appealing visual nature of neuroimaging that could make jurors think that the images corresponded to objective findings. Interestingly, McCabe and Castel (2008)^39^ examined whether brain images affect people’s judgements of scientific credibility. They found that when neuroimages accompanied scientific summaries, the summaries were rated as more scientifically credible than summaries presented alone or paired with graphs. Similar results were obtained by Weisberg et al. (2008)^40^, who demonstrated that jurors become more likely to be convinced by logically irrelevant explanations for behaviours if these explanations are simply expressed in terms of neuroscientific evidence. Although these results do not appear to be replicated in a real forensic setting^41^, they provocatively underline the seductive charm of neuroimaging. This is a clear example of the biasing influence of human involvement on judicial decisions^18^: if sNI evidence is not properly weighed and integrated with other evidence, it is not helpful to the forensic process. This can lead to a potential improper use of sNI in court^42^, where the risk is that consultants are more interested in generating fascinating images of the brain than they are in elucidating their importance for explaining the behaviours that are legally relevant^33^.

For this reason, it is important that neuroscientists understand how to correctly interpret sNI evidence in the process of evaluating insanity^33,34^. Furthermore, it is important for judges and jurors to have sufficient knowledge of the ways in which sNI evidence can be relevant to legal questions and to recognize when it is not. Thus, the aim of the current paper is to present practical guidelines to be used in the delicate process of sNI result interpretation by the following (HEP level of conclusions): (i) forensic neuroscientists, when using neuroimaging as scientific evidence in support of mental incompetence; (ii) judges and jurors, during the sophisticated decision-making process of determining the role of neuroimaging data in support of the criminal liability of defendants. Using the HEP terminology^22,23^, the guidelines aim to enhance intra-rater and inter-rater reliability and minimize bias in conclusions.

Before discussing guidelines, we will introduce relevant concepts via two real-life forensic cases in which neuroscientific evidence was used to support a clinical diagnosis. They were selected mainly due to their similarities: both homicide cases, both likely due to disinhibition, and both with defendants manifesting frontal lobe dysfunction. However, there is one important difference between these cases. In the first, an objective brain lesion was evident, while in the second, there was a subtle alteration in grey matter volume. Thus, in the first case, the forensic discussion focused on the causal link between the brain lesion and the homicide (HEP level of conclusions), while in the second case, the discussion focused both on the investigation of brain abnormalities (HEP level of observations, which will not be discussed in detail) and on the anatomo-clinical correlation between the brain abnormalities and behavioural dysfunction (HEP level of conclusions).

As additional premises, we would like to underline that (i) this paper is focused on the use of structural images only. The introduction of functional images is further complicated by high between-subject variability in the blood oxygenation level-dependent (BOLD) signal, the ecological validity of task performance^43^ and the absence of false-positive distribution maps that can guide result interpretation (this map is available for sNI^4,5^). Thus, the potential application of functional imaging is a topic that falls outside the aims of the current paper. (ii) In this paper, we have purposely decided to present neuroimaging findings in an accessible and charming way in an attempt to give the readers an idea of their charming impact.

## Examples of Real Forensic Application

### Case 1

#### The facts

The defendant was a 40-plus-year-old man charged with murder. In 2014, the man saw a jogger on the street and suddenly stopped and assaulted the woman, who reacted. After having beaten the woman severely, he threw the body off a cliff. The next day, he went back to the scene of the crime and masturbated on the corpse. After ∼3 months, he was interrogated by the police and immediately confessed the murder.

#### The descriptive diagnosis

The defendant was a 40-plus-year-old man who grew up in a rural mountain area. His parents divorced when he was nine. He had completed 5 years of education. He was married and had a son. A few years into the marriage, he suffered from severe head trauma due to a car accident and was hospitalized for 3 months. His marriage lasted <2 years after his accident, and no relationship with the son was maintained. He was unable to keep a job for more than a few months since the accident. Moreover, he developed a serial pattern of criminal sexual behaviour. He was previously jailed for 1 year due to performing frottage and making vituperative comments to women on the street.

At the forensic psychiatric examination, no psychotic symptoms were evident, but the defendant did appear fatuous and avoidant. He seemed to not realize the severity or legal consequences of his behaviour. The neuropsychological examination revealed a borderline IQ, impaired verbal comprehension, and below-average working memory function. The defendant presented with severe frontal executive functioning deficits, in particular in the ability to inhibit automatic answers (as measured using the Stroop test^44^) and behaviours (as measured using the frontal assessment battery^45^). During the neuropsychological evaluation, his behaviour was oppositional; he was easily frustrated with negative feedback and had a tendency to become irritated and verbally aggressive towards the examiner.

#### The brain diagnosis

**Fig. 1:**
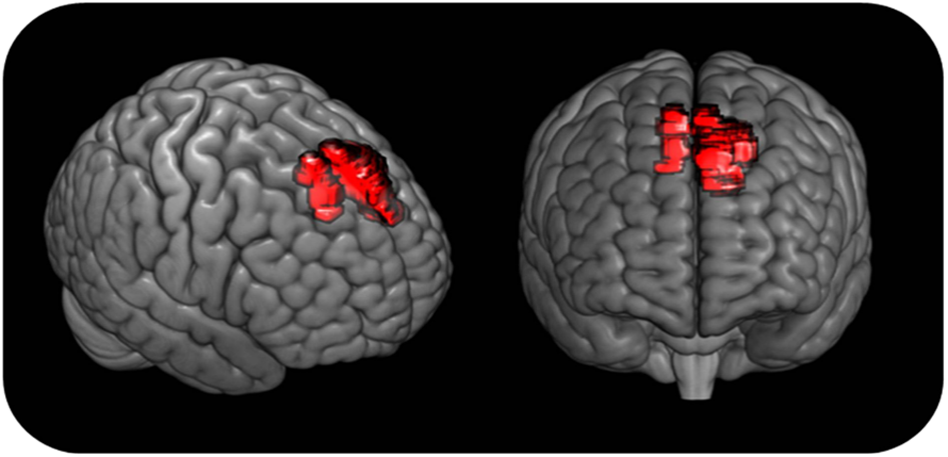
3D reconstruction of the defendant’s exact brain lesion. Based on the CT scan, the brain lesion was manually traced by one of the authors (CS) on the MRIcro template. The traced lesion was then overlaid to the 3D render provided by MRIcroGL software (http://www.cabiatl.com/mricrogl/)

The CT scan (MRI could not be acquired due to the defendant’s pacemaker) showed a brain lesion, which was likely a result of the head trauma that had occurred 10 years prior. The lesion was detected bilaterally in the mesial prefrontal cortex, measuring 12.29 cm^3^ (Fig. 1).

#### Interpretation of the results

According to the judge’s expert, the neuroimaging data in this criminal case were fundamental in explaining the constellation of symptoms that characterized the defendant’s history, as well as the crimino-dynamics that led to the homicide. From the defendant’s anamnesis, it is evident that he had undergone a “behavioural fracture” from being a man with a normal life, marriage, and job to exhibiting sexually depraved/disinhibited behaviour and an inability to maintain normal relationships or jobs. Moreover, his sexual behaviour was carried out in a disorganized and risky manner. The man used to perform frottage on women in broad daylight, and in the case of the murder, the aggression occurred near a main road with a high probability of being seen.

Critically, the defendant manifested additional behavioural symptoms that were a strong indication of an affected ability *to understand* and *to will*. According to the Italian penal code, these two capabilities are the legal basis of the insanity plea. Regarding the defendant’s ability to understand, he seemed not to realize the legal consequences of his actions; he did not understand the risk of being caught (supported by his return to the scene of the crime), and he manifested a deficit in moral reasoning. Regarding the defendant’s ability to will, his personal history clearly revealed previous aggressions towards women, previous outbreaks of violence, and repeated inability to inhibit sexual urges. All these symptoms strongly indicated pathology of the prefrontal cortex. Even more interestingly, he manifested symptoms that were legally irrelevant *per se* but strongly support frontal lobe dysfunction through the “convergence of evidence” principle. For instance, he was unable to inhibit non-sexual impulses, as emerged from the neuropsychological examination and from his verbally aggressive behaviour during said evaluations.

The presence of the brain lesion is also important from the crimino-dynamic prospective: after the traumatic brain injury, the defendant manifested sexually deviant behaviours mainly characterized by frotteurism. Sexual alterations after traumatic brain injury are well known in the literature^46,47^. Thus, there is a strong cause for causality between the brain lesion and the alteration in sexual behaviour. However, this is not sufficient to explain the murder. The defendant’s violent behaviour may be explained by his easy frustration in response to negative feedback (the woman’s refusal). This frustration was followed by aggressive and disinhibited behaviour (the strike), which found its equivalent in verbal aggression towards the examiner during the neuropsychological examination. Again, disinhibition is strongly suggestive of frontal lobe dysfunction^48–50^. Notably, the defendant’s manifest insensitivity to the consequences of behaviour corroborated the idea of frontal lobe deficits^51^. Thus, a strong anatomo-clinical correlation between the brain lesions and pathological symptoms of the defendant is evident.

In summary, in this case, the judge’s expert concluded that the constellation of symptoms, legally relevant or not, could be explained by the presence and location of the traumatic brain lesion. The criteria for mental insanity are grounded in the defendant’s behaviour, and neuroimaging findings provide a useful explanation for them. The judge concluded that the defendant should thus be considered not responsible for his behaviour.

#### What if I get it wrong?

Gianfranco Stevanin was an Italian serial killer^43^. He suffocated six prostitutes during extreme sexual activities and then dismembered and buried their bodies. The man had suffered a severe head injury resulting in a bilateral lesion of the frontal lobes (no images are available). From the time of the brain injury, he manifested asphyxiophilic tendencies^52^. Because the frontal lobes are the brain regions responsible for impulse control and inhibition, the man was initially declared not responsible for the murders. According to the defence thesis, as a result of the bilateral frontal lobe lesion, the man was unable to inhibit sexual activity once it began. Corroborating this hypothesis, the neuropsychological evaluation revealed the presence of many symptoms that clearly indicate frontal lobe dysfunction: disadvantageous decision-making, impaired impulse inhibition and easy irritability. In contrast, his behaviour after the murders, e.g., the dismemberment and concealment of the dead bodies, could not be explained by a deficit in impulse inhibition. Critically, it was then discovered that Gianfranco Stevanin used to practise the very same extreme sexual activities with his fiancée, without killing her. These actions provided unequivocal evidence discrediting the claim that the man could not interrupt his sexual impulses. In this case, the presence of the brain injury could not be used to mitigate the man’s responsibility for his crimes. Gianfranco Stevanin’s case is of outstanding importance to exemplify that, even in the presence of a clear brain lesion, the interpretation of its impact on behaviour is “somewhat subjective” (human element^18^) and that clinicians should consider all clinical and behavioural information to correctly interpret the meaning of the imaging findings.

### Case 2

#### The facts

The defendant was a 24-year-old woman, JF, who was charged with murder for smothering her newborn child to death immediately after delivery^31^. She then wrapped the infant’s body in a towel and hid it inside a suitcase. The defendant later claimed that the newborn child was ‘born dead’ due to drug abstinence syndrome (see below).

#### The descriptive diagnosis

Anamnestic information revealed that the defendant started to heavily smoke cigarettes at the age of eleven. She had a well-documented history of multidrug abuse, as well as alcohol abuse, since the age of thirteen. She had become pregnant during a party and did not interrupt her drug use during pregnancy.

The forensic psychiatric examination revealed that the defendant had a personality profile characterized by antisocial features such as a history of illegal behaviour, sensation seeking, familial conflict, lack of sensitivity, rejection of conventional standards, poor response to threatening situations and a willingness to forgo careful consideration of alternative solutions to problems. The neuropsychological evaluation revealed impulsivity, i.e., inability to inhibit the prepotent response (as measured by means of the Hayling test^53^); a deficit in planning (as measured by means of the Tower of London^54^); and deficits in emotional attribution and in identifying violations of social norms (as measured by the emotion attribution task^55^ and social situation task^56^).

#### The brain diagnosis

**Fig. 2:**
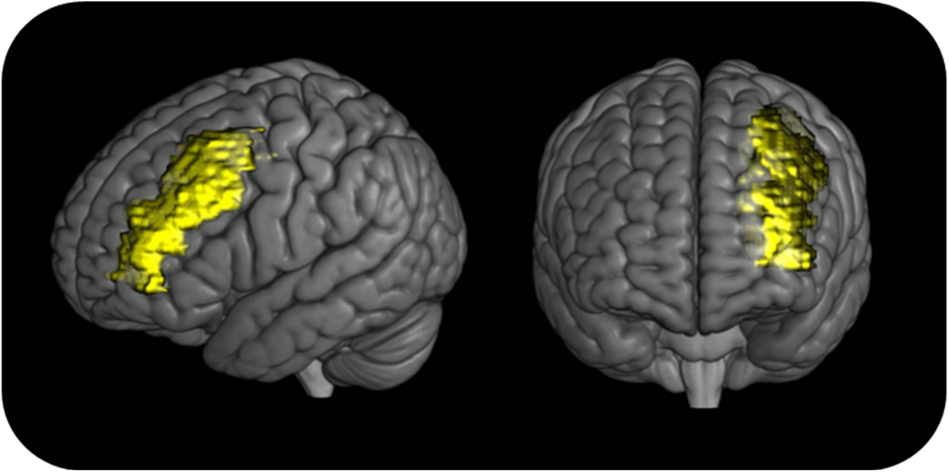
3D reconstruction of the localization of JF’s brain deficiency in grey matter volume. In the image, the exact extent of JF’s abnormality is not represented, but only the location. In the reference paper^31^, the grey matter loss is reported to be localized in the middle frontal gyrus. For this image, a binary mask on the whole middle frontal gyrus was created using PickAtlas (http://fmri.wfubmc.edu/software/pickatlas) and was then overlaid onto the 3D render provided by MRIcroGL software (http://www.cabiatl.com/mricrogl/)

The structural MRI of the defendant was compared with the MRI of healthy women using voxel-based morphometry (VBM), a neuroimaging technique that highlights subtle structural anatomical abnormalities^35,36^. The analysis revealed a reduced grey matter volume in the left prefrontal cortex in JF relative to the control group^31^ (Fig. 2).

#### Interpretation of the results

According to the defence consultant, the sNI data in this case were fundamental in explaining the constellation of symptoms that characterized the defendant’s history. Indeed, the site of the brain alteration (i.e., the frontal lobe) has a strong anatomo-clinical correlation with the descriptive diagnosis, which can be summarized as frontal syndrome, as the defendant manifested symptoms ascribable to frontal lobe dysfunction, particularly impulsivity^57^, a deficit in planning, a deficit in automatic response inhibition^58,59^ and difficulty in emotional attribution^60^, as well as alteration of the personality characterized by high scores on the borderline^61^, depression^62^ and substance abuse^63^ subscales. Furthermore, according to the false-positive distribution map^3^, the decrease in grey matter in the dorsolateral prefrontal cortex (DLPFC) in a single individual compared to the control group has only a 0.5–5% probability of being a false positive.

The presence of the brain alteration in the frontal lobe is also important from the crimino-dynamic prospective: the symptoms identified in JF showed that she had a reduced capacity to control her behaviour. This was extremely relevant for the evaluation of the prosecutor’s hypothesis, according to which JF *impulsively* smothered the newborn to death. Thus, the clinically identified pathological features are causally linked to the crime, providing the basis for an insanity defence^31^. JF’s behavioural reports, accompanied by reduced grey matter in the frontal lobe, provided “hard” biological evidence for the defence’s case.

In summary, the defence consultants concluded that the constellation of behavioural and neuropsychological symptoms manifested by JF were in strong anatomo-clinical correlation with the brain abnormalities and were causally linked to the crime. According to the defence consultants, JF should be considered not responsible for her behaviour because she was unable to inhibit her impulses.

#### What if I get it wrong?

**Fig. 3:**
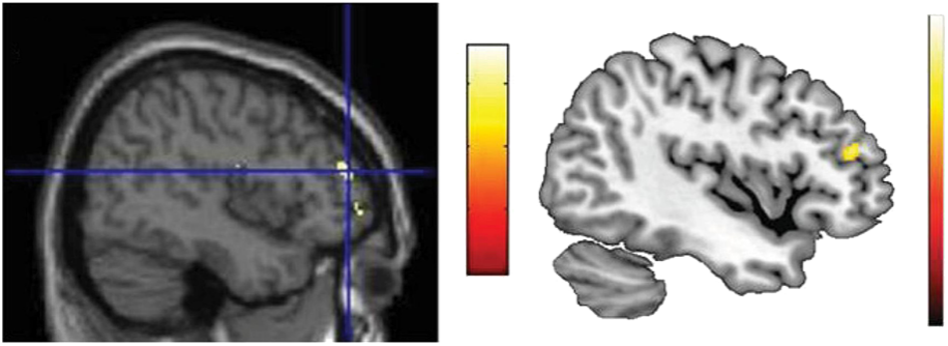
VBM results. Reprinted from ref. ^4^. Left panel: VBM result of the defendant’s brain. Right panel: VBM results in binge drinking; image adapted from ref. ^64^

A woman was charged with murder for running over a man with her car. She underwent psychiatric and psychological evaluations to understand her state of mind at the moment of the homicide. The descriptive diagnosis gave a negative result. The defendant’s brain MRI, analysed using VBM methodology, revealed an increase in grey matter in the DLPFC, particularly in the middle frontal gyrus (Fig. 3, left panel). In the absence of any descriptive diagnosis, this result can be interpreted only according to the literature. Since an increase in DLPFC grey matter has been linked to binge drinking^64^ (Fig. 3, right panel), it would be tempting to infer that the defendant was a binge drinker and that the accident occurred because she was drunk.

This conclusion and reasoning are invalid, since inferring the presence of altered mental states (being a binge drinker) from the presence of a brain alteration (increased grey matter in the DLPFC) is a logical fallacy called reverse inference^65^. This fallacy is particularly relevant in neuroimaging studies, where behaviours are subserved by complex brain networks and each brain region is consequently involved in more than one behaviour^66^. Furthermore, according to the false-positive distribution map^6^, an increase in grey matter in the DLPFC in a single individual compared to the control group has up to a 23% chance of being a false positive. Thus, this result, which has no clinical correlate (i.e., is not in anatomo-clinical correlation with the descriptive diagnosis), is likely to be a false positive result, probably reflecting normal variability in neuroanatomy rather than any brain pathology^6^. Indeed, this case was fictional, and the VBM results reported highlighted the grey matter increase in the brain of one of the authors (CS) compared to a control group.

## Discussion

In the current paper, we aim to provide clear guidelines for the correct use of sNI in court. Our opinion is that sNI should support behavioural findings to reduce controversies in court. Both of the described cases clearly demonstrate the importance of using neuroimaging findings strictly in conjunction with behavioural evidence. Based on the danger of incorrectly claiming the presence or absence of a causal relationship between neuroimaging findings and behaviour, we believe that careful behavioural assessment remains more reliable than neuroimaging in ascertaining the relevant mental states that form the basis of legal criteria.

While it is certainly true that neuroimaging is a powerful tool whose potential for misuse can potentially cause deleterious effects in a legal setting^67^, we believe it is a mistake to deny its potential helpful applications a priori^12,68^. Although neuroscientists often commit logical errors when presenting isolated neuroscientific findings^33,67^, we believe a similar logical error is committed by ignoring neuroscientific data^69^. It is our opinion that the legal and scientific communities should work together to create guidelines that ensure the correct application of neuroscientific techniques and the appropriate interpretation of neuroscientific findings. In the following paragraphs, we will discuss general rules for the use of neuroimaging. The correct application of these rules is extremely important to allow group-to-individual inference, a problem that neuroscientists recognize and are trying to solve^2–6,67^. Finally, we will propose guidelines that might be useful to neuroscientists, lawyers, judges and jurors. These rules and guidelines will help to increase within- and between-expert reliability and to reduce within- and between-expert bias in expert testimony conclusions^22,23^.

### On the role of neuroimaging in court

*Rule number 1. Neuroimaging results should be coupled with behavioural findings*. Neuroimaging results assume a meaning only when coupled with a clear descriptive diagnosis, i.e., with clear symptoms manifested by the defendant. Indeed, without a clear descriptive diagnosis, the neuroimaging findings cannot be interpreted, as described in the paragraph “What if I get it wrong?” of case 2. This rule has been violated in numerous cases reported in the literature. One example is the famous case of Weinstein, where the defence attorney used the neuroimaging findings of a subarachnoid cyst in the frontal lobe to support the insanity of the defendant^67,70^. Although the presence of arachnoid cysts has been associated with atypical psychosis^71^, no one tested for the presence of psychosis in the defendant or his inhibitory abilities, moral reasoning, etc. We recognize that this rule may open the door to biased neuroimaging results because there is a motive to couple them with behavioural findings. Although this is true, this potential bias lies at the observation level of HEP and can be overcome by blinding the experts to the descriptive diagnosis of the defendant^20,72^. Rule number 1 applies mainly to wider forensic reasoning (HEP conclusions), when the attorneys need to integrate evidence.

*Rule number 2. The criminal behaviour cannot be considered a symptom*. Anatomo-clinical correlation can be assessed between a brain region and cognitive function but cannot be assessed between a brain region and criminal behaviour, as there is no specific brain region involved in complex behaviours such as criminality or violence. Despite investigation of this topic in recent studies^73^, evidence suggests that complex behaviours are supported by highly complex brain networks^73,74^. Complex behaviours can be broken down into contributing cognitive functions, i.e., an irresistible impulse murder may be partially explained by a deficit in impulse control. Thus, the hypothetical brain abnormality must be put in anatomo-clinical correlation with the lack of impulse control, which should be demonstrated clinically (rule number 1) and, if possible, by evidence beyond the criminal act. For example, we recently studied a case of acquired paedophilia^12,68^ and claimed that the defendant was unable to inhibit his impulses. To support this hypothesis, we referred to the man’s recently emerged kleptomania, a fact that is irrelevant to paedophilia but relevant to impulse control. Rule number 2 was violated in the case of Weinstein, as the defence attorney argued the anatomo-clinical correlation between the cyst in the frontal lobe and the violent murder, using the violent murder as a symptom of the cyst^67,70^. This rule would help to minimize the adversarial allegiance bias^17,23^, which refers to the tendency of experts to reach conclusions supporting the side that retained them.

*Rule number 3. Not every brain abnormality leads to behavioural symptoms*. The correlation between brain function and criminal behaviour is far from perfect. Although violent behaviour is more likely in patients with frontal lobe deficits than in patients with (for example) parietal or occipital deficits, not every patient with frontal lobe damage manifests violent behaviour. In addition to the famous case of Phineas Gage, the poet Guillaume Apollinaire should be considered. After being wounded in the right temporal lobe during World War I, Apollinaire became unaffectionate and indifferent to his girlfriend, his personality became defiant and nostalgic, and his work became darker—but he never manifested aggressive behaviour^75^. Indeed, the brain is incredibly plastic, and different clinical conditions demonstrate that even large brain lesions may not lead to important behavioural deficits. An example is Rasmussen encephalopathy, a rare inflammatory brain disorder leading to the death of one hemisphere^76^. To date, the only known effective treatment is a hemispherectomy. The literature reveals that if a hemispherectomy is performed during childhood, patients may not have severe neurologic sequelae^76^. Rule number 3 has been violated in the case of Gianfranco Stevanin, as the defence attorney wrongly ascribed his violent behaviour to the frontal lobe deficit. This rule would help in minimizing confirmation bias^20^, which describes the tendency of experts to reach conclusions that confirm what they already believe to be true.

*Rule number 4*. *Do not reason backwards*. To infer the presence of an altered mental state from the presence of brain pathology is a *reverse inference*. Reverse inferences are a logical fallacy that should be avoided^16,65^. The correct neuroscientific reasoning is the following: the defendant behaviourally manifested difficulties in behavioural control; the defendant also has a brain lesion in the frontal lobe; thus, the brain lesion might account for the lack of behavioural control. Assuming the presence of a psychiatric or neurological disorder from brain images is dangerous both from the clinical (i.e., wrong drug treatment) and forensic (i.e., incorrect conclusion on insanity) points of view. Rule number 4 was violated in both Weinstein’s case and the case described in the “What if I get it wrong?” paragraph of case 2. As with rule 1, rule 4 can be applied to both the observation and conclusion levels of HEP. One way to mitigate the bias of backwards reasoning at the HEP level of observation is to avoid providing neuroimaging experts with irrelevant case information, which might cause cognitive contamination^20^. One way to mitigate the risk of bias at the HEP level of conclusions is to request neuroimaging investigation only when the defendant presents with clear psychiatric or cognitive symptoms, as explained in the following guidelines.

### Practical guidelines to avoid misinterpretation of results

**Fig. 4:**
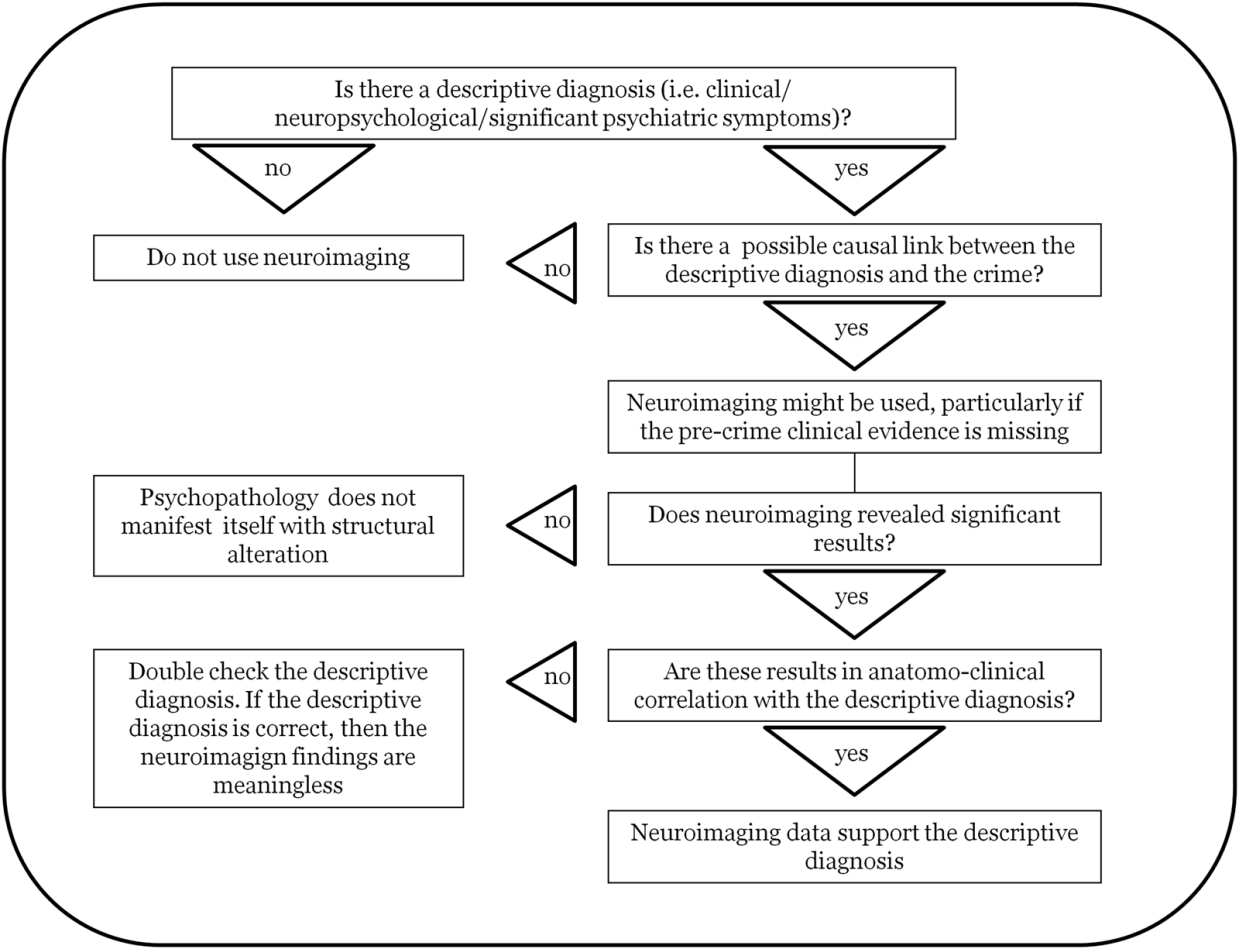
**Flow chart summarizing the guidelines for the correct use of neuroimaging in a forensic setting**

In the current paragraph, we will operationalize the rules in practical guidelines for the correct use of neuroimaging in court. The aim of these guidelines is to mitigate the impact of cognitive biases^18^ and the misinterpretation of the neuroimaging results often presented in forensic trials^67^. In particular, the correct application of these guidelines will help to minimize both within- and between-expert biases in conclusions^23^, including the adversarial allegiance bias^17,23^. The guidelines are graphically represented in Fig. 4.

- *Provide a descriptive diagnosis*, i.e., describe the symptoms manifested by the defendant, as behavioural evidence is the gold standard for determining functional impairment. The descriptive diagnosis should be carried out, when possible, with standardized neuropsychological tests including indexes for controlling malingering and exaggeration (e.g., symptom validity testing^77^). If the descriptive diagnosis is negative, as in the fourth case described in the current paper, then any accompanying sNI is useless and should thus be excluded. If the descriptive diagnosis is positive, investigation can proceed to step 2.
- *Assess the causal link between the symptoms and the crime*. The causal link between altered state of mind (i.e., symptoms) and crime^78^ has not been discussed in the current paper, as it was beyond the scope of our topic. However, it is important to remember that in the forensic setting, the symptoms of the defendant are important if they can be causally linked to the *actus reus*. For instance, impulse disinhibition is of great relevance in the context of the irresistible impulse to murder, while it is not relevant in the context of premeditation. If there is no causal link between the descriptive diagnosis and the crime, then sNI should not be used, as there is no way for eventual results to be interpreted. If there is a causal link between the descriptive diagnosis and the crime, sNI evidence might be useful, particularly if pre-crime clinical evidence is missing.
- *Clarify whether the neuroimaging techniques highlight significant results*. In the case that neuroimaging techniques produce no results, it can be concluded that the psychopathology does not manifest itself with structural alterations. It is important to remember that the presence/absence of psychopathology can be deduced only by behavioural findings (point a); thus, a negative sNI result does not imply the absence of psychopathology. The identification of neural correlates of psychiatric or personality disorders is highly complicated and may be more evident at the functional level than at the structural level^79^. Of course, if malingering is suspected, negative neuroimaging results could prompt a search for additional behavioural evidence. If sNI results are positive, the investigation can proceed to step d.
- *Use brain imaging to assess anatomo-clinical correlation*. Are the neuroimaging results in anatomo-clinical correlation with the descriptive diagnosis? Although neuroscientists agree that complex behaviours are the result of a dynamic network of interconnected brain regions^80,81^, it is also true that some cognitive functions rely strongly on a specific brain region: for instance, behavioural control requires the correct functioning of the prefrontal cortex^82^. Thus, a frontal lobe deficit should be expected in a defendant manifesting behavioural impulse disinhibition. If there is no anatomo-clinical correlation, then we suggest that the descriptive diagnosis be checked again. If the descriptive diagnosis is correct, then the sNI results, despite their presence, are not relevant to explain the defendant’s behaviour, as in the case of Gianfranco Stevanin. This may be attributable to brain plasticity. Only if there is anatomo-clinical correlation can the sNI data be used to support the descriptive diagnosis.

### Limitations

It is important to acknowledge some potential limitations of the current paper. First, we are referring only to structural neuroimaging findings, as we purposely omit discussion of functional neuroimaging for the reasons described in the introduction. Thus, our rules and guidelines cannot be generalized to the use of functional images in court. The use of functional imaging in this setting is, in our opinion, still premature. Second, we neglected to discuss the within- and between-expert biases at the observation level of the HEP model^22,23^, as there are different ways in which images can be analysed, and the literature on the topic is so wide that it would have been impossible to summarize in the current paper. We are aware that apparent unreliability at the level of conclusions may lie at the level of observation, and this would require a different intervention^23^. Future studies are needed to explore this topic.

## Conclusions

In the current paper, we used real forensic cases to highlight how neuroimaging results should be interpreted when used as an additional instrument to reduce cognitive bias at the HEP conclusion level^22,23^ and thus reduce controversies in the assessment of insanity. Through a detailed analysis of two cases and an explanation of errors that one may commit if results are not correctly interpreted, we offered four general rules for the use of neuroimaging in court. Finally, we proposed guidelines for the correct use of neuroimaging in a forensic setting. Our aim was to provide neuroscientists with a practical guide when facing the difficulty of using neuroimaging as scientific evidence in support of a mental incompetence claim.

Although the present study focuses on the use of sNI in a forensic setting, the insights from the current analysis, as well as the rules and guidelines defined, might be of interest for a broad range of neuroscientists.

Indeed:

- We are claiming that sNI results not coupled with behavioural findings are meaningless. Thus, this paper may be of interest for disentangling the controversies on the general role of sNI results^12,67,69^.
- The rules and guidelines proposed could be adopted outside forensic contexts and could be applied to individual cases, for instance, in clinical contexts^2–6^.
- The current study provides important, practical and exploitable guidelines that can ultimately be of immediate use to clinicians and neuroscientists who are interested in the real-world application of neuroscientific findings.
